# Research on Equivalent Scale Analysis for On-Orbit Assembly of Ultra-Large Space Structures

**DOI:** 10.3390/ma18245508

**Published:** 2025-12-08

**Authors:** Dayu Zhang, Xiaofei Ma, Yang Li, Zexing Yu, Ruiwen Guo, Wenjin Liu, Sicheng Wang, Yongbo Ye

**Affiliations:** 1 China Academy of Space Technology (Xi’an), Xi’an 710100, China; dyzhang@mail.nwpu.edu.cn (D.Z.);; 2 Xi’an Institute of Space Ratio Technology, Xi’an 710100, China; yuzexing@mail.nwpu.edu.cn; 3 School of Astronautics, Northwestern Polytechnical University, Xi’an 710072, China

**Keywords:** ultra-large space structure, on-orbit assembly, scale-down equivalent, natural frequency, static deformation

## Abstract

Ultra-large structures serve as core aerospace equipment for missions such as Earth observation and deep space exploration. With dimensions reaching hundreds of meters or even kilometers, they require advanced technologies, including on-orbit assembly, modular integration, and robot-assisted construction, to achieve high-precision structural formation and stable operation. For on-orbit assembly of these structures, critical attention must be paid to their inherent vibration characteristics to evaluate on-orbit service stiffness and stability. Additionally, the static deformation behavior during assembly must be examined to assess the impact of assembly loads on overall structural deformation and surface accuracy. To efficiently evaluate the above-mentioned characteristics, an equivalent scale analysis method for the on-orbit assembly of space-based megastructures is established. Through theoretical modelling, it establishes scaling relationships between mechanical properties—such as structural natural vibration and static deformation—and module diameter dimensions. The numerical results indicate that halving the module diameter results in the natural frequency of the assembled structure increasing by about four times and the static deformation decreasing by about eight times, in agreement with the scaling law. This method enables accurate inference of the full-scale structure’s on-orbit mechanical behavior, thereby facilitating precise evaluation of typical mechanical characteristics during ultra-large structure on-orbit assembly.

## 1. Introduction

On-orbit assembly is a crucial method for constructing large and ultra-large space structures such as space stations, large satellite antennas, large-aperture space telescopes, and space solar power stations. It represents a game-changing disruptive technology [1]. The assembly of ultra-large space structures on-orbit is characterized by being highly flexible, having ultra-low natural frequencies, and exhibiting incremental configuration growth. Considering that the scale of on-orbit assembly for ultra-large space structures can reach hundreds of meters, with individual modules unfolding to nearly ten meters in size, conducting full-scale ground experiments is impractical. Therefore, establishing equivalence relationships between on-orbit modules and scaled-down ground modules is essential. This involves comprehensively verifying the equivalence of dynamics and deformation between the two types of modules. The primary challenge lies in establishing dynamic and deformation equivalence between scaled ground modules and actual on-orbit modules to validate and extrapolate the on-orbit dynamic behavior of antennas [2,3,4].

For ultra-large space structures at the assembly stage, the assembly process is characterized by a large number of modules, extensive variations in system configuration and parameters during assembly, and, consequently, more complex dynamic modelling and analysis. Existing research on the dynamics of on-orbit assembly modules has predominantly focused on coupled dynamics and collision dynamics during the assembly process. Chen et al. systematically investigated two scenarios: self-assembly of space structures [5] and robotic assembly of ultra-large space structures [6]. Employing both natural coordinate methods and absolute node coordinate methods for dynamic modelling, they calculated the dynamic responses during the assembly process. Regarding the orbit–attitude–structure coupled dynamics model for assembling large-scale structures in space, employing a global coordinate system to describe large-scale orbital motion, small-scale attitude motion, and structural vibration, as well as micro-scale contact/collision, presents accuracy loss issues [7]. Wu et al. [8,9,10] proposed ‘expansion-based’ and ‘extraction-based’ structural dynamic modelling approaches tailored to the on-orbit assembly of large space solar power stations, thereby avoiding extensive repetitive modelling work and significantly enhancing modelling and simulation efficiency. They further established an orbital–attitude–structural coupled dynamics model for the assembly process, analyzing its dynamic characteristics under varying pitch attitude angles and attitude control schemes. Rong et al. [11] employed an absolute node coordinate method to describe flexible truss structures, achieving precise simulation of the on-orbit assembly dynamics for large-scale space power stations. Li et al. [12] addressed the on-orbit assembly of the OMEGA space solar power station. Employing Tschauner–Hempel equations and finite element modelling, they derived dynamic equations for assembly modules, investigating multiple scenarios involving single- and dual-robot assembly. However, their modelling approach relied on traditional orbital, attitude, and structural descriptions without accounting for coupling effects. Concerning the study of special dynamic coupling mechanisms in space-based ultra-large structures, Deng et al. [13,14] established integrated dynamic equations under the Hamiltonian framework using the absolute node coordinate method. Employing symplectic methods, they achieved high-precision solutions and analyses of dynamic responses, investigating quasi-static deformation and structural vibrations in sun-tracking beams induced by gravitational gradients. They further analyzed beam resonance phenomena caused by geometric nonlinearity and gravitational gradients. Furthermore, they [15] proposed an absolute node coordinate method and natural coordinate method based on local translational coordinate systems, decoupling large-scale orbital motion from other motions to enhance numerical simulation accuracy and efficiency, though collision motions during assembly were not considered. Based on similarity theory and multi-flexible body system sensitivity analysis, Shan et al. [16] proposed a scaled equivalent dynamic modelling method for space multi-flexible body systems. By designing a similarity-scaled model through solving fundamental dimensional scales, this approach circumvents the cumbersome analysis of similarity criteria for complex systems. Various space agencies [17,18,19] around the world have conducted in-orbit experiments on relevant research results to verify emerging space robotic arm technology. Before launching, space robotic arms must undergo ground verification of their related technologies. Therefore, various research institutions have developed a large number of ground experimental platforms to promote the research and development of space robotic arms.

With regards to ultra-large structures, they are assembled in circles or sectors (like fan-shaped) by using robots. Regardless of the assembly method chosen, the structure develops into a cantilever beam structure as the assembly process progresses. Especially when the number of assembly modules exceeds three, the out-of-plane deformation, particularly the bending deformation, will intensify. Therefore, there is a need to pay special attention to the effects of bending deformation and structural vibration on the assembly process. In summary, current research on the dynamics of large-scale structures’ on-orbit assembly has predominantly focused on the coupling analysis of small-scale attitude motions, structural vibrations, and micro-scale collision motions during the assembly process. There remains a scarcity of studies examining the equivalence of dynamics and deformation between full-scale modules assembled on-orbit and scaled-down assembly modules tested on the ground. This paper investigates the dynamic equivalence relationship between full-scale on-orbit assembly structures and ground-based scaled-down modules, thereby providing an equivalent scaled modelling and analysis method for large-module assembly antennas.

## 2. Composition of Space-Based Ultra-Large On-Orbit Assembly Structural Systems

### 2.1. System Composition

The ultra-large weight and dimensions of space-based structures make on-orbit assembly the most effective approach for constructing high-performance satellite-mounted ultra-large parabolic structures. Such structures must satisfy conflicting technical requirements, including large dimensions, low stiffness, and high precision. The assembly process involves complex dynamic phenomena such as incremental configuration development, attitude–orbit–structure coupling, and contact collisions, presenting significant challenges in structural design and construction. The overall assembly scheme design constitutes a critical foundational issue. It is imperative to consider mission requirements, maneuverability capabilities, and on-orbit resource support capacity. Analyzing the deployment complexity, structural stiffness upon deployment, and stowage volume of different module types enables the selection of module configurations and topological structure design, ultimately forming an on-orbit implementable configuration scheme for the ultra-large space structure.

**Figure 1:**
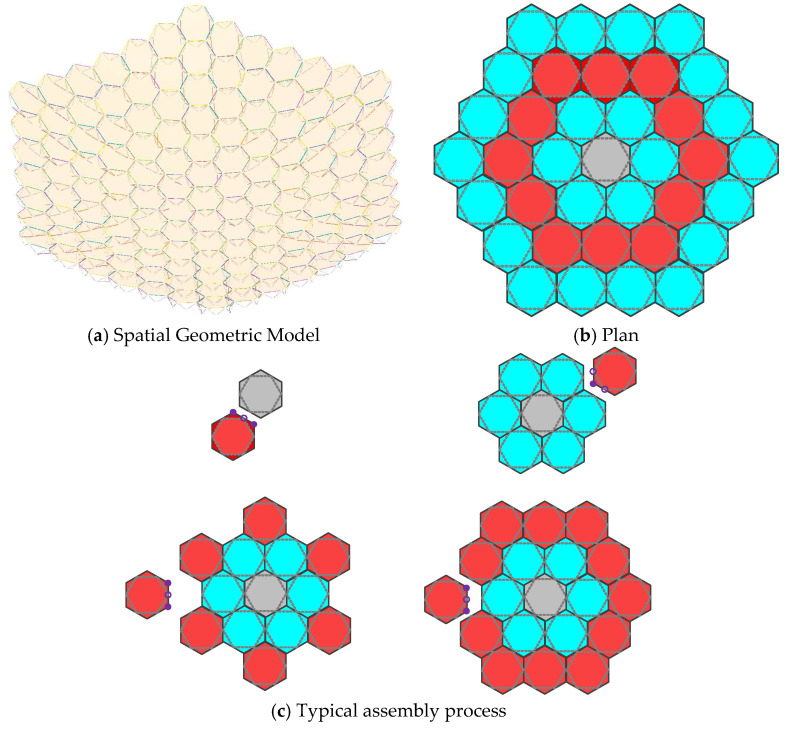
Composition of Space Ultra-Large On-Orbit Assembly Structural Systems and Typical Assembly Processes.

The subject of this paper is a spatial ultra-large structure, as illustrated in Figure 1. The spatial ultra-large structure comprises multiple hexagonal modules. As all units employ identical geometric dimensions and cannot be assembled into a spherical surface, a projection method is employed for the topological design of the module units. The aperture and quantity of module units can be determined based on parameters such as the spatial structure’s aperture and focal length. The module units exhibit excellent structural rigidity and high mesh surface forming precision. The inter-module docking mechanisms offer good operability, high connection strength and rigidity, and high docking accuracy, enabling rapid, efficient, stable, and reliable connections between adjacent modules.

### 2.2. Assembly Module Structure

**Figure 2:**
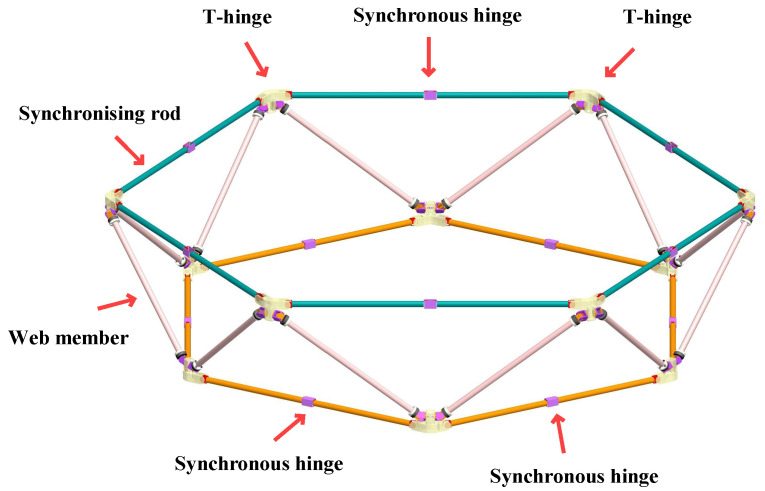
Composition of Space Ultra-Large On-orbit Assembly Structural Systems.

The on-orbit assembly of ultra-large space structures relies on modular units, making the structural and mechanical design of deployable module units crucial for achieving on-orbit assembly. A deployable module unit with retraction, deployment, and docking capabilities was designed and developed. The module unit comprises a truss structure and a module retraction locking mechanism, with the truss structure based on hexagonal deployable modules. The deployable module is shown schematically in Figure 2.

The modular truss comprises synchronous hinges, T-hinges, synchronous rods, and auxiliary rods connecting the upper and lower T-hinges. During deployment, the upper and lower synchronous rods move in opposite directions, driven by torsion springs or coil springs within the synchronous hinges, simultaneously rotating the auxiliary rods around the T-hinges. T-hinges are employed at the nodes on the upper and lower surfaces of the module. In the retracted state, both surfaces form hexagons of identical shape and size, with the hexagon dimensions determined by the T-hinges. The upper and lower drive rods assume a vertical orientation upon retraction, with their retracted dimensions dictated by the drive hinges.

The drive rod and auxiliary rod of the module are connected via a T-hinge. During module retraction and extension, the auxiliary rod rotates about the T-hinge. Given the limited rotation angle of the auxiliary rod about the lower surface T-hinge, to simplify the mechanism design and reduce structural weight, the auxiliary rod may be connected to the lower surface T-hinge using a spherical plain bearing to accommodate small-angle rotation.

## 3. Equivalent Analysis Calculation Process for Ultra-Large Assembled Structures

Since the ultra-large structure exhibits typical slender rod-like geometric characteristics, this paper first employs beam–rod structural analysis methods to model and analyze the natural vibration characteristics and static deformation properties of the module structure. It further establishes the proportional relationship between the structure’s natural frequencies and static deformation quantities and its dimensional scale, ultimately deriving a scaling law between the key mechanical properties of the assembled module and its caliber.

This section focuses on the computational process for analyzing the equivalent natural frequencies of large-scale assembled structures. First, establish the geometric model of the modules. Given that the module structure exhibits typical slender rod-like geometric characteristics, beam elements are employed to discretize and model the structural members using finite element analysis. Material properties and cross-sectional shapes are assigned to the members to reflect the elastic deformation characteristics of the structure.

Multiple module models are established based on their spatial geometric positions. Assembly between adjacent modules is achieved through mating surfaces, each configured with three mating nodes. To ensure continuous stiffness transfer at connection points after spatial structure assembly, degree-of-freedom constraints must be applied to the mating nodes. The application of degree-of-freedom constraints between paired mating nodes on adjacent module mating surfaces is expressed as:

(1) $$\begin{array}{l} U_{s}(x,y,z)=U_{m}(x,y,z)\\ UR_{s}(x,y,z)=UR_{m}(x,y,z) \end{array}$$

In the formula, the subscript m denotes the master node, and the subscript s denotes the slave node. By binding the degrees of freedom, the translational and rotational degrees of freedom of the slave node are consistently aligned with those of the master node, thereby achieving an ideal rigid connection between the various modules. The upper model information is applied as the initial state to the main solver for mechanical analysis.

For the nature frequency analysis, set the six corner points of the central module in the assembled structure as fixed boundary conditions, meaning both translational and rotational degrees of freedom are set to zero. Perform an eigenfrequency analysis and a static deformation analysis on the structure, as described below.

Based on the finite element model of the assembled structure and its mass and stiffness matrices, the system’s intrinsic vibration equation is formulated as follows:

(2) $$M\ddot{u}+Ku=0$$

where M denotes the system mass matrix, K denotes the system stiffness matrix, and u denotes the system displacement vector. The principal vibration of this system may be defined as:

(3) u=ϕsin(ωt+φ)

Substituting Formula (2) into Formula (3) yields the following system of algebraic homogeneous equations:

(4) $$(K-\omega^{2}M)\phi =0$$

The necessary and sufficient condition for the existence of a non-zero solution ϕ to the above system of equations is that the determinant of the coefficient matrix is zero, that is:

(5) $$\left|K-\omega^{2}M\right|=0$$

Formula (5) is referred to as the characteristic equation of the system, where $\omega^{2}$ is termed the eigenvalue or characteristic root, whose value depends solely on physical parameters such as the system’s stiffness and mass. Arrange all solutions $\omega_{i}$ of Formula (5) in ascending order of their arithmetic square roots, corresponding to the i-th natural frequency of the system. Substituting $\omega_{i}$ into Formula (4) yields the corresponding eigenvector $\phi_{i}$, representing the i-th mode shape of the system. Using the above method, the first six natural frequencies and their corresponding mode shapes of the assembled structure are calculated, completing the analysis of the system’s natural vibration characteristics.

Since the module frame behaves as a slender structure with a fixed boundary at one end during operation, the assembled configuration can be reasonably idealized as a cantilever beam under such boundary constraints. The governing equation for free vibration of the beam structure, considering the assumptions of small deformation and linear elastic materials, is derived as shown in Formula (6), where EI, L, ρ, and A represent the bending stiffness, length, density, and cross-sectional area of the beam structure, respectively.

(6) $$EI\frac{\partial^{4}y}{\partial x^{4}}+\rho A\frac{\partial^{2}y}{\partial t^{2}}=0$$

The principal vibration of the beam can be assumed as:

(7) y(x,t)=Y(x)sin(ωt)

where ω is the angular frequency. By substituting Formula (7) into the governing equation:

(8) $$\frac{d^{4}Y}{dx^{4}}-\beta^{4}Y=0$$

Define:

$$\begin{array}{cc} \beta^{4}=\frac{\omega^{2}}{a^{2}}, & \begin{array}{c} a^{2}=\frac{EI}{\rho A} \end{array} \end{array}$$

The general solution to this differential equation is

(9) $$Y(x)=C_{1}\cos\beta x+C_{2}\sin\beta x+C_{3}\cosh\beta x+C_{4}\sinh\beta x$$

where $C_{1},C_{2},C_{3},C_{4}$ are constants determined by boundary conditions.

For a cantilever beam structure, the deflection and slope at its fixed end are zero, which is

(10) $$\begin{array}{cc} Y(0)=0, & Y^{\prime }(0)=0 \end{array}$$

The bending moment and shear force at the other end of the cantilever beam are zero, which is

(11) $$\begin{array}{cc} Y^{\prime \prime }(L)=0, & Y^{\prime \prime \prime }(L)=0 \end{array}$$

At location x = 0, substitute Formula (10) into the Formula (9) and simplify:

(12) $$\begin{array}{l} C_{1}+C_{3}=0\\ C_{2}+C_{4}=0 \end{array}$$

At location x = L, combining Formulas (9), (11) and (12), we obtain

(13) $$\left\{\begin{array}{c} (\cos\beta L+\cosh\beta L)C_{1}+(\sin\beta L+\sinh\beta L)C_{2}=0\\ (\sin\beta L-\sinh\beta L)C_{1}-(\cos\beta L+\cosh\beta L)C_{2}=0 \end{array}\right.$$

For non-trivial solutions, the determinant of the coefficient matrix must be zero:

(14) $$\left|\begin{array}{cc} \cos\beta L+\cosh\beta L & \sin\beta L+\sinh\beta L\\ \sin\beta L-\sinh\beta L & -(\cos\beta L+\cosh\beta L) \end{array}\right|=0$$

Compute the determinant:

(15) cosβL·coshβL=−1

The roots of Formula (14) are

(16) $$\beta_{1}L=1.875,\;\beta_{2}L=4.694,\;\beta_{3}L=7.855,\;\beta_{i}L\approx (r-\frac{1}{2})\pi \;i\geq 4$$

The natural frequency of cantilever beam structure are

(17) $$\omega_{i}=\beta_{i}^{2}a={\left(\beta_{i}L\right)}^{2}\frac{1}{L^{2}}\sqrt{\frac{EI}{\rho A}}$$

Hence:

(18) $$\begin{array}{l} \omega_{1}=\frac{3.516}{L^{2}}\sqrt{\frac{EI}{\rho A}},\;\omega_{2}=\frac{22.035}{L^{2}}\sqrt{\frac{EI}{\rho A}},\;\\ \omega_{3}=\frac{61.697}{L^{2}}\sqrt{\frac{EI}{\rho A}},\;\omega_{i}=\frac{{\left[(2r-1)\pi /2\right]}^{2}}{L^{2}}\sqrt{\frac{EI}{\rho A}}\;(i\geq 4) \end{array}$$

The natural frequency $\omega_{i}$ of each mode in the cantilever beam structure is inversely proportional to its length $L^{2}$. The relationship between the natural frequency of the cantilever beam structure and its structural length dimension has thus been established. Without altering the material properties, cross-sectional shape, or geometric configuration of the module members, a change in module diameter D results in a corresponding alteration to the equivalent cantilever beam length L of the assembled structure. Based on the established relationship between the natural frequency of cantilever beam structures and their dimensional length, a proportional relationship for the natural frequencies of assembled structures with different diameters is further derived and expanded. A scaling law is established as shown in Formula (19):

(19) $$\frac{U_{a,max}}{U_{b,max}}=\frac{L_{a}^{3}}{L_{b}^{3}}=\frac{D_{a}^{3}}{D_{b}^{3}}$$

From the above work, it is evident that compared to increasing the aperture of a modular antenna by a factor of n, the equivalent cantilever beam length of the assembled structure increases by a factor of n. Under identical static loads, the maximum static deformation of the system increases by a factor of $n^{3}$. Computational models for assembled structures of varying apertures were established to validate the scaling relationship between the assembled module aperture and structural static deformation.

**Figure 3:**
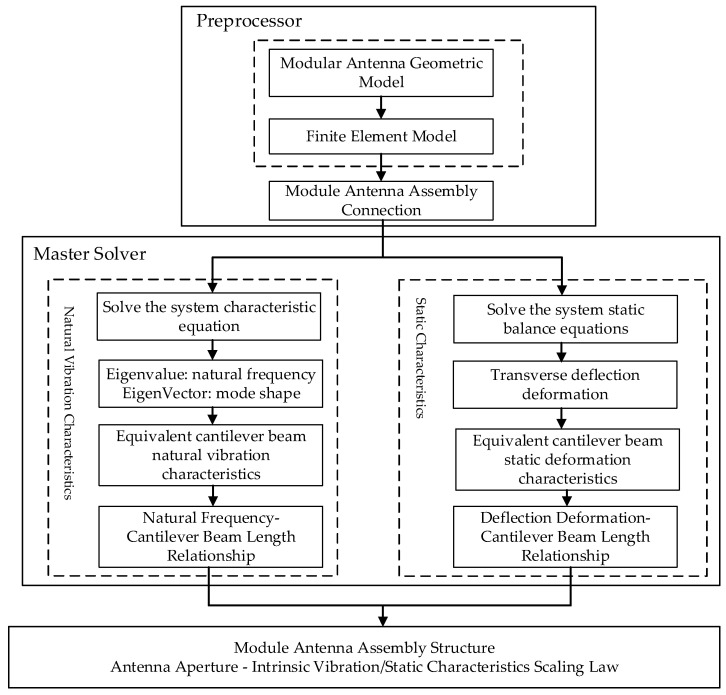
Flowchart of the Equivalent Scale Modelling Analysis Method.

Procedures for the equivalent scaled modelling analysis method of large-scale assembly structures are shown in Figure 3.

It is worth noting that the proposed scaling law is established under the assumptions of linear elasticity and small deformations, which are consistent with the conditions of this kind of structure in its operational state. More complex scenarios—such as those involving geometric non-linearity—fall beyond the scope of this study, and the applicability of the scaling law under such conditions needs further investigation.

## 4. Equivalent Analysis Numerical Simulation

### 4.1. Numerical Simulation Model and Boundary Conditions

**Figure 4:**
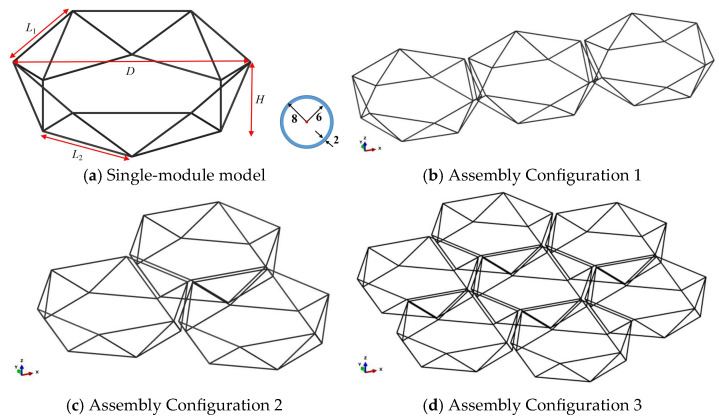
Modular assembly model.

To further validate the scaling relationship between the established module assembly structure’s natural vibration characteristics, static deformation properties, and module diameter, in this section, we establish corresponding numerical simulation models. Three assembly configurations are investigated, with three module diameters (9.6 m, 4.8 m, and 2.4 m) selected for computational comparison under each configuration. The assembled module model studied is shown in Figure 4. Each module features a polyhedral structure with hexagonal upper and lower frames connected by diagonal struts. Assembly configuration 1 consists of three modules connected sequentially along the x-direction. Configuration 2 involves three modules connected in a fan-shaped arrangement. Configuration 3 comprises six modules connected circumferentially around a central module.

In different assembly configurations, adjacent modules are joined via mating surfaces, each equipped with three mating nodes. To ensure continuous stiffness transfer at the connection points after module assembly, the mating nodes must be paired and subjected to degrees-of-freedom constraints, which has already been discussed in the previous Formula (1). The finite element model of the assembly structure was constructed using a two-node beam element (B31) in the commercial finite element software code Abaqus 2020. The total number of elements was selected to be 17,600 for each module through a mesh sensitivity study. Fixed boundary conditions were applied at the six corner points of the hexagonal frame at the base of the central module, where both translational and rotational degrees of freedom were constrained to zero. The concentrated load was applied at the free end of the assembly structure for static deformation analysis. Natural vibration characteristics and static deformation characteristics were investigated for three assembly configurations and three module diameters, respectively.

### 4.2. Natural Vibration Characteristics

**Figure 5:**
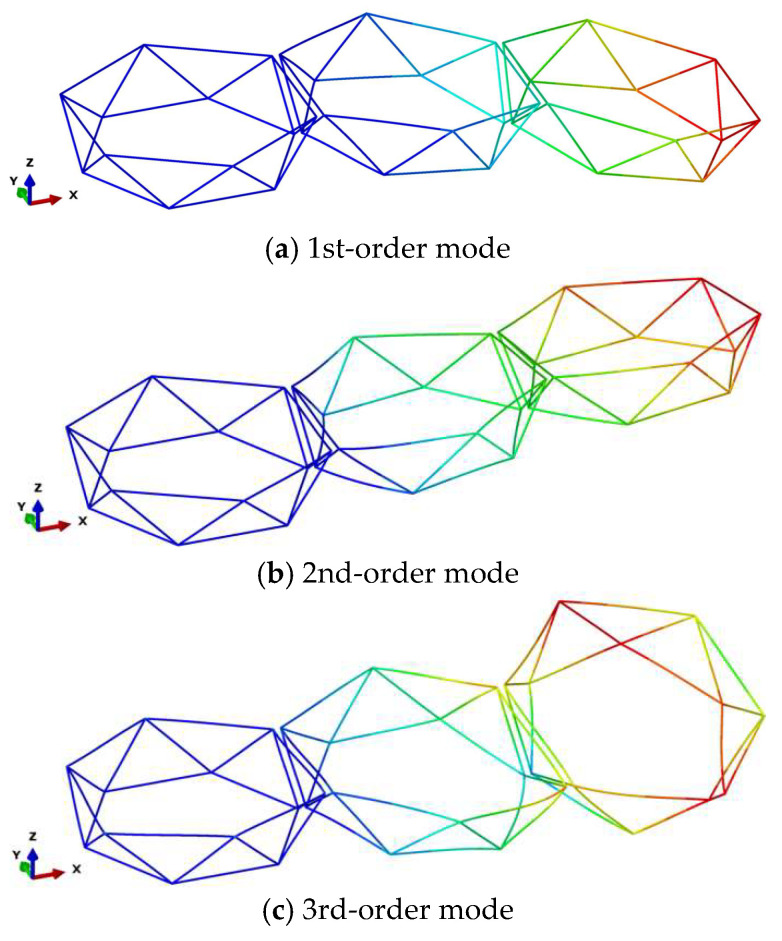
Assembly Configuration 1: First Three Modal Shapes.

The first three modal shapes of assembly configuration 1 are shown in Figure 5. The first-order modal shape represents bending deformation about the y-axis, the second-order shape represents bending deformation about the z-axis, and the third-order shape represents a coupled bending–torsional deformation. It is worth noting that under this assembly configuration, altering the diameter of individual modules yields consistent modal shapes, though the corresponding natural frequencies differ for each order.

**Figure 6:**
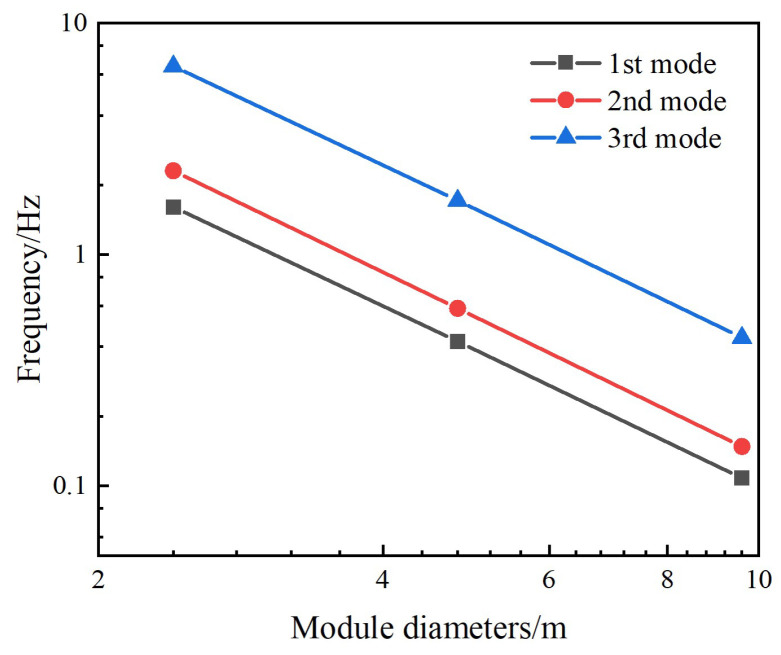
Assembly Configuration 1: The variation of the first three natural frequencies with the module diameter.

Figure 6 compares the first three natural frequencies of different module diameters in assembly configuration 1. The data are presented in logarithmic form to visually present the scaling law between the natural frequency and the diameter.

The more detailed data of the first six natural frequencies in assembly configuration 1 are listed in Table 1. Using the 9.6-metre diameter as the reference, when the module diameter is reduced to half and one-quarter of the original diameter, each natural frequency increases by approximately 3.9 and 15 times, respectively. This aligns well with the scaling law established in Section 2 between the natural frequencies of the assembled module structure and the reduced module diameter.

**Figure 7:**
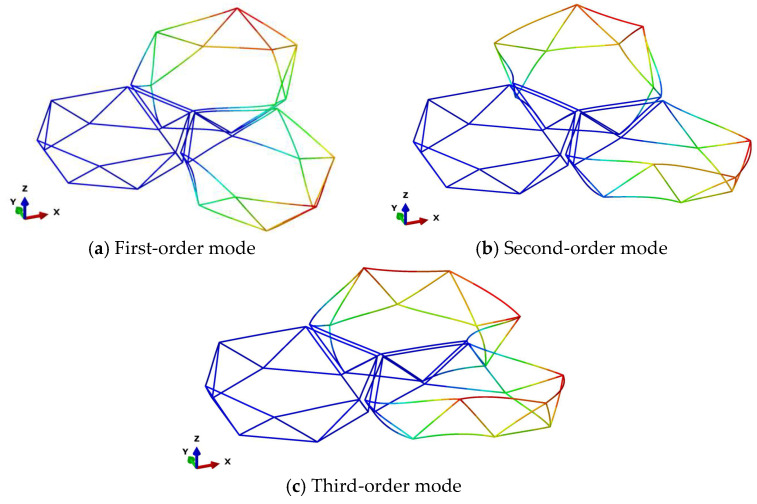
Assembly Configuration 2: First Three Modal Shapes.

**Figure 8:**
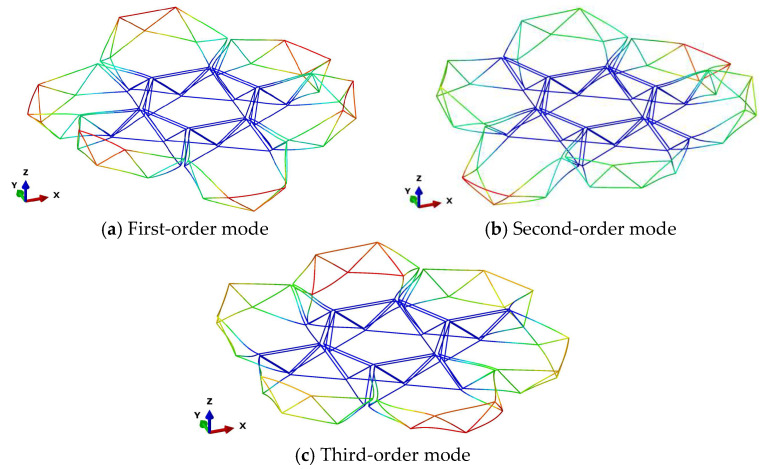
Assembly Configuration 3: First Three Modal Shapes.

Figure 7 presents the first three modal shapes for assembly configuration 2. The first-order modal shape exhibits bending deformation about the y-axis, the second-order modal shape demonstrates bending deformation about the z-axis, and the third-order modal shape displays tensile deformation along the x-axis. Figure 8 presents the first three modal shapes for assembly configuration 3. The first and second modal shapes represent alternating bending deformations of the peripheral modules along the z-axis, while the third modal shape depicts overall bending deformation of the peripheral modules about the z-axis. Within the same assembly configuration, when the diameter of individual modules varies, the geometric principles of the assembled structure remain unchanged; consequently, the corresponding modal shapes remain consistent.

**Figure 9:**
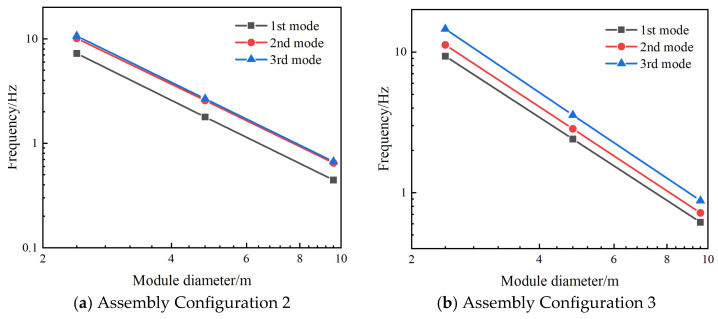
The variation of the first three natural frequencies with the module diameter under assembly configurations 2 and 3.

Figure 9 compares the first three natural frequencies of different module diameters in assembly configurations 2 and 3. The variation of the natural frequency with the diameter is approximately a straight line in the logarithmic plot. It can also be analyzed from Figure 9 that the natural frequency is inversely proportional to the square of the diameter. More specific values can be seen in Table 2 and Table 3 for assembly configurations 2 and 3, respectively. Consistent with the pattern observed in Table 1, when the diameter of a single module increases by a factor of n within the same assembly configuration, each corresponding natural frequency of the overall structure approximates 1/n^2^. Numerical calculations further validate the scaling law for the natural frequencies of the modular assembly structure relative to module diameter proposed in Section 2, while also demonstrating the applicability of this scaling law across different assembly configurations.

### 4.3. Static Deformation Characteristics

**Figure 10:**
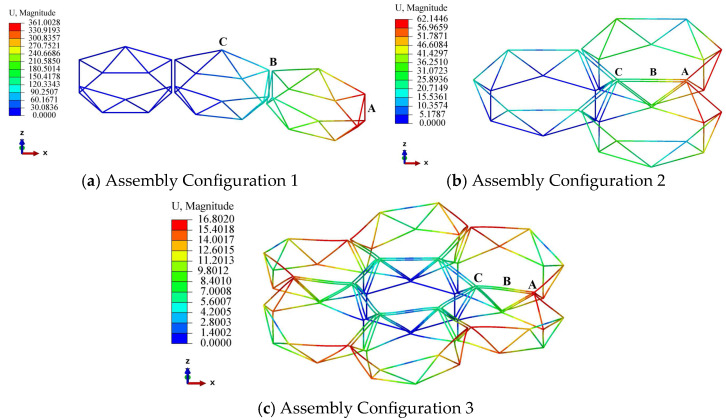
Static Deformation Patterns of Assembled Structures under Three Assembly Configurations.

In this subsection, we analyze the static deformation characteristics of the assembled structure under assembly loads. By altering the diameter of individual modules and comparing static deformation values, the scaled-down law established in Section 2 is validated. During numerical calculations, a concentrated load of 10 N was applied downward along the z-axis to the free end of the assembled structure. Figure 10 illustrates the static deformation profiles under three assembly configurations. Under the concentrated load, Assembly Configurations 1 and 2 exhibited pronounced bending deformation. In Assembly Configuration 3, the loaded side underwent downward bending deformation along the z-axis, while the circumferentially symmetrical opposite side exhibited upward bending deformation along the z-axis.

**Figure 11:**
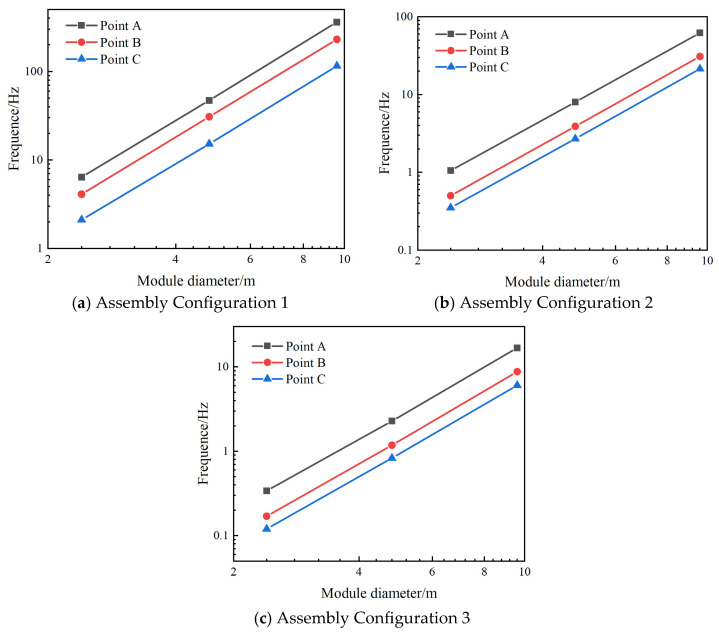
The static deformation of three critical locations with the variation of module diameters.

For each assembly configuration, static deformation calculations were performed while maintaining identical external loads but altering the individual module diameters within the assembly. The resulting deformation values at three critical locations—*A*, *B*, and *C*—were extracted for comparison. Figure 11 compares the static deformation of three critical locations with the variation of module diameters in assembly configurations 1, 2, and 3. The horizontal and vertical data in the figure are all plotted in logarithmic form, and through the slope of the curve, the scaling law between deformation and diameter can be clearly seen.

More specific static deformation values of assembly configurations 1, 2 and 3 are summarized and listed in Table 4. Analysis of the data reveals that when the module aperture is doubled and quadrupled, the static deformation at the critical locations for assembly configurations 1 and 2 increases by approximately 7.6 times and 56 times, respectively. For assembly configuration 3, the static deformation at the critical locations increases by approximately 7.3 times and 50 times, respectively. These proportional relationships approximate 23-fold and 43-fold increases, indicating that the static deformation of the assembled structure is proportional to the cube of the antenna aperture. This aligns with the scaling law proposed in Section 2. However, it should be noted that the scaling law in Section 2 was derived using cantilever beam assumptions, whereas the actual deformation behavior of the loaded module assembly structure does not entirely correspond to that of a cantilever beam. Therefore, the proportional relationship obtained from the numerical simulations exhibits some deviation from the scaling law in Section 2. Nevertheless, it generally reflects the static deformation behavior of assembled antennas with different aperture sizes.

The equivalent scale analysis aims to improve the design of the scaled model through dynamic or static analysis methods, so that the results of the scaled experiments can better reveal the dynamic characteristics of the original model. This research is important for large-scale space solar power stations with dimensions reaching kilometer levels, ultra-large space payloads (such as space-based solar wings or robotic arms), and space scientific exploration infrastructures (such as space-based telescopes) [20,21,22,23]. The existing ground experimental systems and methods for space structures are unable to meet the full-scale ground experimental requirements for the on-orbit assembly process of a large space structure. The four aspects of dimension, frequency, deformation, and control should be examined in the future [2,16,24], and it is suggested to establish a scaled experimental ground test using the equivalent theory and dimensional analysis method to the local–global mapping algorithm, ensuring the reliability of the on-orbit assembly of the large space structure.

## 5. Conclusions

The ground-based testing of ultra-large space structures presents significant challenges, which are effectively mitigated by the proposed scaling law. Through experimental data obtained from small-scale prototypes, it enables rapid and accurate prediction of the mechanical properties of full-scale structures, thereby providing technical support for assessing the on-orbit service status of ultra-large space structures.

(1) This paper investigates the on-orbit assembly of ultra-large space structures, establishing dynamic and deformation equivalence between on-orbit modules and ground-based scaled models. This framework enables reliable analysis and validation of large-scale assemblies and accurate assessment of mechanical properties, including natural vibrations and static deformation.
(2) Regarding natural frequency analysis, increasing the module aperture D by a factor of n reduces each natural frequency to 1/n^2^ of the original design. Using a 9.6 m aperture as baseline, halving and quartering the aperture increases each natural frequency by 3.9 and 15 times, respectively, in good agreement with the scaling law.
(3) For static deformation, enlarging the module aperture by a factor of n increases the equivalent cantilever length by n, resulting in a maximum deformation that scales with n^3^ under identical static loads. Thus, the static deformation of the assembled structure is proportional to the cube of the module aperture.

The findings of this study provide valuable guidance and reference for accurately assessing the mechanical properties—such as natural vibrations and static deformation—of ultra-large space structures during on-orbit assembly.

## Figures and Tables

**Table 1:** Comparison of Natural Vibration Frequencies for Different Module Bores in Assembly Configuration 1 (Hz).

Module Bore	1st Mode	2nd Mode	3rd Mode	4th Mode	5th Mode	6th Mode
9.6 m	0.108	0.148	0.438	0.512	0.590	0.671
4.8 m	0.422	0.586	1.714	2.019	2.360	2.679
2.4 m	1.608	2.303	6.521	7.940	9.420	10.670

**Table 2:** Comparison of Natural Vibration Frequencies for Different Module Bores in Assembly Configuration 2 (Hz).

Module Bore	1st Mode	2nd Mode	3rd Mode	4th Mode	5th Mode	6th Mode
9.6 m	0.44514	0.64717	0.66748	0.74270	1.0293	1.1714
4.8 m	1.7833	2.5670	2.6673	2.9646	4.1088	4.7212
2.4 m	7.2487	10.100	10.638	11.759	16.337	18.978

**Table 3:** Comparison of Natural Vibration Frequencies for Different Module Bores in Assembly Configuration 3 (Hz).

Module Bore	1st Mode	2nd Mode	3rd Mode	4th Mode	5th Mode	6th Mode
9.6 m	0.61515	0.71697	0.87698	0.89783	0.95803	1.3704
4.8 m	2.4038	2.8454	3.5600	3.6863	3.8298	5.4359
2.4 m	9.3461	11.230	14.577	15.232	15.291	21.356

**Table 4:** Comparison of Static Deformation at Critical Locations for Different Module Diameters Under Three Assembly Configurations.

	Module Bore	Deformation at Point A/mm	Deformation at Point B/mm	Deformation at Point C/mm
Assembly configuration 1	9.6 m	361.0	230.1	115.4
	4.8 m	47.1	30.8	15.2
	2.4 m	6.4	4.1	2.1
Assembly configuration 2	9.6 m	62.1	30.9	21.4
	4.8 m	8.0	3.9	2.7
	2.4 m	1.05	0.50	0.35
Assembly configuration 3	9.6 m	16.82	8.78	6.05
	4.8 m	2.28	1.18	0.83
	2.4 m	0.34	0.17	0.12

## Data Availability

The original contributions presented in this study are included in the article. Further inquiries can be directed to the corresponding authors.
